# Determinants of Antiretroviral Therapy Adherence among Women in Southern Malawi: Healthcare Providers' Perspectives

**DOI:** 10.1155/2014/489370

**Published:** 2014-12-28

**Authors:** Ogbochi McKinney, Naomi N. Modeste, Jerry W. Lee, Peter C. Gleason, Gisele Maynard-Tucker

**Affiliations:** ^1^School of Public Health, Loma Linda University, 24951 North Circle Drive, Nichol Hall, Loma Linda, CA 92350, USA; ^2^University of California Los Angeles (UCLA) Center for the Study of Women, Los Angeles, CA 90095, USA

## Abstract

*Background*. The purpose of this study was to explore healthcare providers' perspectives on antiretroviral (ART) adherence in two ART clinics in southern Malawi. Nonadherence to ART is a significant hindrance to the success of HIV/AIDS treatment.* Methods*. A one-on-one semistructured interview was conducted with eight healthcare providers in two ART clinics in rural and urban southern Malawi. The interviews were focused on factors facilitating or hindering ART adherence and strategies to improve adherence. Interviews were audio-recorded, transcribed, and content-analyzed with the use of the constant comparison approach.* Results*. Of the eight participants, 63% were between the ages of 20 and 30 years and 37% were HIV counselors. Factors facilitating adherence include patients' belief and knowledge, HIV/AIDS education, and a supportive network. Barriers to adherence include discrimination, nondisclosure of HIV status, food insecurity, medication side effects, religion, misinformation, and staff and drug shortages. Strategies to improve adherence were identified by participants to include nutritional/food supplementation for malnourished or undernourished patients and patient counseling.* Conclusions*. There is a need for collaborative efforts between healthcare providers, patients, and faith-based organizations to identify and address hindrances and facilitators to patients' adherence. Further research is needed to develop strategies addressing religion, staff, and drug shortages.

## 1. Introduction

Between 2009 and 2011, the number of HIV-positive persons receiving antiretroviral therapy (ART) increased by 59% in sub-Saharan Africa [1]. Malawian women aged 15–49 have the highest rate of HIV prevalence at 13% compared with 8% for men. Females make up about 51% of adults with HIV in Malawi [2–4]. In 2007, an estimated 163 sites in Malawi were reported as providing ART to HIV patients [5]. Three years later, Joint United Nations Program on HIV/AIDS (UNAIDS) and World Health Organization (WHO) [1] reported that 228, 478 HIV-positive individuals were receiving ART treatment compared with 96,000 in 2006. In 2010, about 57% of HIV-positive individuals were reported to have ART coverage, a 29% increase in coverage from the previous year [6]. Adherence is defined by WHO as the “degree to which individual's behavior regarding taking medications, following a diet, and performing lifestyle changes follows agreed recommendations from a healthcare provider” [7]. To minimize the progression of HIV to AIDS, decrease mortality, and maintain long-term suppression of viral load, a minimum adherence level of 95% is required [8, 9]. The Malawi National Human Immunodeficiency Virus (HIV)/Acquired Immunodeficiency Syndrome (AIDS) Commission recognizes proper adherence as greater than or equal to 95% [10]. However, the adherence rate in Malawi is below the recommended 95% [11].

The Malawian government has implemented a plan to increase adherence to ART by having patients identify a guardian who reminds, facilitates, and supports patients in taking their medications regularly [12]. The guardians and patients are required to attend the first group counseling session, where they are provided with education about ART and the importance of adherence [12]. To assess whether this strategy was successful, HIV/AIDS treatment centers were asked to measure adherence at each visit by self-reporting missed doses, which were then validated by a physical pill count [12]. Malamulo Hospital (rural) and the Queen Elizabeth Central Hospital (QECH) ART Clinic (urban) are among those treatment centers that provide HIV/AIDS related services to Malawian patients. However, no known study has assessed or compared adherence of patients attending these two facilities. Greater understanding of some of the factors that influence ART adherence among this population is relevant and could help in the development of culturally appropriate interventions to increase ART adherence, which could improve outcomes for those on ART medications. We conducted an exploratory study using in-depth interviews with healthcare providers to understand their perceptions of HIV patients' adherence to ART.

## 2. Methods and Materials

### 2.1. Study Site and Participants

This qualitative study was conducted with purposive sample of eight healthcare providers at two ART clinics located in rural and urban hospitals in southern Malawi. The rural ART clinic is operated in a faith-based hospital owned by Seventh-Day Adventist (SDA) Christians while the second ART clinic located in the urban city of Blantyre is government-owned and operated. Both ART clinics provide inpatient and outpatient HIV/AIDS services to all individuals with HIV/AIDS regardless of age, income, or religious background. However, most of the patients come primarily from low-income and low educational backgrounds. The native populations of the region we studied speak Chichewa as their primary language.

Healthcare providers who have worked directly with HIV patients on ART for at least 1 year in the selected clinics were eligible to participate. Research assistants who were knowledgeable about each study site recruited healthcare providers. The targeted population was solicited for the study during their break hours and at staff meetings. The chief medical officer at the rural hospital and the head nurse at the urban hospital introduced researchers to potential participants at the weekly staff meeting. The purpose of the study was to explore healthcare providers' perceptions of their patients' adherence behaviors and their perceptions of determinants of adherence to ART. After recruitment, healthcare providers were informed that their participation in the study was voluntary and told that refusal would not affect their relationship with the hospital or affect their job. Eight healthcare providers were selected to participate in the study from the two ART clinics. Malamulo ART Clinic has just five staff members and of those five, we interviewed the four who were on duty during our study period. The same applied to QECH ART Clinic. Healthcare providers who agreed to take part in the study were offered verbal informed consent. Loma Linda University Institutional Review Board (IRB) and University of Malawi College of Medicine Research and Ethical Committee (COMREC) approved the study.

### 2.2. Data Collection and Materials

The research group consisted of one experienced researcher and two trained research assistants. Training on how to conduct interviews was provided to all facilitators including appropriate protocols and obtaining informed consent from participants. The interviews were pilot-tested in a similar clinic away from both research sites. The interview questions, although only semistructured, were guided by the theory of planned behavior using these criteria: (a) access and barriers, (b) treatment adherence, (c) challenges in ART service provision, (c) beliefs and norms, and (d) strategies used to increase adherence to ART. Interviews were conducted at each healthcare providers' office in Chichewa (Malawi's national language) or English as desired by each provider. The interviews were audio-recorded using a digital recorder and transcribed verbatim by a research assistant who had not participated in the interview process. Transcribed interviews conducted in Chichewa were translated into English by two other researchers, and the translations were then sent to an independent researcher for comparison. The researcher concluded that the content was the same with very few variations in the sentence structure; therefore, we proceeded with data analysis, which provided a more accurate English translation of the answers participants gave to the research questions.

### 2.3. Data Analysis

Data were analyzed manually using content and thematic analysis, organized into reoccurring themes, and then categorized before analysis. The grounded theory approach was used for analysis. The first phase of coding consisted of deductive codes (codes grounded in theory) that were drawn from the interview guide and research questions. The second phase consisted of inductive coding, which was used to identify patterns in the interviews; then categories were established to identify additional themes, patterns, and categories that emerged from the data [13]. Deductive coding is the use of theoretical framework to corroborate or refute ideas, while inductive coding uses data to generate ideas [14].

Data were summarized through descriptive text-based summaries and data display matrices [15, 16]. Verbatim quotes were selected to exemplify key findings, themes, and codes. The constant comparative method [17], which codes and analyzes qualitative data to develop categories and compare data [18], was used to compare and contrast participants' responses between and within interviews. This approach also involves comparison within a single interview, comparison between interviews within the same group, comparison of interviews from different groups, and comparison in pairs [19]. Axial coding was used to identify characteristics for each concept developed before defining the concept [19]. Conventional content analysis [20] was used to code and classify data, to make sense of the data that were collected, and to highlight relevant messages and findings.

## 3. Results and Discussion

Characteristics of the interviewees are summarized in Table 1. Eight healthcare providers (four females and four males) were included in the study. Six interviewees had at least 7–10 years of experience working with HIV/AIDS patients, while two interviewees had less than 7 years of experience. The majority of interviewees were between 20 and 30 (63%) years old and 37% (*n* = 3) of participants interviewed were HIV counselors.

All of the interviewees cited barriers affecting adherence on the individual level to include nondisclosure of HIV status to others, discrimination, lack of access to food, and medication side effects. Barriers affecting adherence on the provider level were staff and drug shortages. Factors facilitating adherence included HIV/AIDS education, patients' belief and knowledge of ART outcomes, and support groups, which consisted of peers, family, friends, and other patients on ART. Strategies to improve adherence included use of nutritional supplements and counseling patients on how to take the medication and other issues relating to the medication.

### 3.1. Disclosure and Discrimination

The majority of the interviewees cited disclosure of HIV status and stigma as factors contributing to nonadherence. Seven out of eight healthcare providers stated that patients go to ART clinics far from home to avoid friends and family because they fear discovery of their HIV status. The following quotes illustrate participants' response to what they perceived as factors hindering adherence:* “The issue of stigma and discrimination is still there. This is manifested by the fact that we are seeing people who are not of our catchment area, and when you probe more from them, they tell us that we [sic] are not comfortable going to our respective Health Centers because we do not want people whom we stay close with, to know that we are HIV positive.”* Discrimination also affects patients' willingness to disclose their HIV status, as illustrated in the following quote,* “Like most of them, they do not disclose that they are going to the clinic to get ARTs. They just say, ‘I am going to the hospital to see a doctor,' they do not want to be sidelined.” *In addition, providers stated that patients do not disclose their status to their employer because of fear of discrimination, and this can affect their adherence to ART. This is illustrated in the following quotes,* “I can say that we note something from patients who did not disclose anything to their immediate boss. If their immediate boss does not know, it becomes very difficult for them to go to the hospital for refill. The boss may not let them leave the office until they finish some specific jobs because the boss did not know that the patient wanted to go to the clinic to refill his/her medication.” *According to the majority of providers,some patients fear disclosing to their boss because they think they may be fired if their status is known.* “They think that maybe if their bosses know their status, they will fire them.”*

Although healthcare providers advocate for disclosure of HIV status to family members, more than half of the providers stated that some of the women fear disclosing their HIV status to their spouses because their husbands may leave them.* “There are some families whereby a woman is tested and is positive but she has not disclosed to the husband, but the time for her to take her medications comes in the presence of her husband and she misses the drug because she is afraid of her husband seeing her take the medication.” *Another provider stated,* “There are some women who haven't disclosed to their husbands because they are afraid that if they disclose, the marriage might end.” *Patients who disclosed their status to spouses/family members tend to adhere better, as illustrated in the following quote:* “[For] those that their partners know about their HIV status, their adherence is good, because they do support each other in the family.”*

### 3.2. Lack of Access to Food

Constant lack of access to food was cited by all healthcare providers interviewed as one of the reasons patients did not adhere to their ART. They also reported that they overheard patients discussing with other patients how they feel when they take their medication without food, and this may have contributed to their inconsistency in taking their medication as directed. One healthcare provider stated,* “They feel hungrier when they take the medication. In addition, I hear some of the patients with the new drug saying, ‘We feel hungry when we take the ARTs.' Some patients do not take the medicines frequently on a daily basis as advised because they do not have enough food.” *Another provider stated,* “If they do not have anything to eat, they also skip medications, because they have the belief that medications are supposed to be taken after taking food. People still have the mentality that they cannot take medications if they haven't eaten.” *One of the providers stated that when she asked patients,* “Why you skipped your dose, sometimes the patients would say because ‘I did not eat.' But most of the patients and clients feel that taking the drugs on an empty stomach will have an effect, so that compromises adherence as well because if they have to look for food it means time for taking the pill is over, and the pill is supposed to be taken twice a day. They may even miss one of the doses.”* Two out of eight providers stated that women were being told to eat before taking their medication,* “We teach them about the food guidelines for the medication. They were told to eat before they take the medications.”* Another stated,* “…they told us that they were told to eat every time they take their medications*.*”* This contradicts what two providers said when asked if lack of food keeps the women from taking their ART. One provider stated,* “We were telling them that they could swallow a pill without taking breakfast and then eat later.”* Another stated,* “Of course we had these cases back in the days but now it is very rare, because we taught them that they do not need to eat for the medicines to work properly in their bodies, so there is no point of not taking the medicines even if they do not have food…today, hunger cannot stop them from taking the medicine.”*

### 3.3. Medication Side Effects

Medication side effects were identified as a possible barrier to ART adherence. All of the healthcare providers interviewed stated that although patients are currently being transferred from the old regimen (Tenofovir) to the new regimen (Efavirenz), side effects are still a common reason their patients reported missing medication doses. The following quotes further illustrate providers' response to patients' experience with medication side effects:* “If they have side effects, sometimes they just decide on their own to stop the drugs instead of coming to the hospital and seeking treatment on the problems that they are having due to the drugs*.* Like last week, I was seeing a certain man who was telling me that he was feeling dizzy so he was opting to take the drugs on alternate days, take one today then tomorrow he does not take the one he took today. He was coming back with pill count that wasn't expected so upon probing more, we discovered that he was taking the drugs on alternate days because he was afraid of the side effects.”* When asked why he thinks patients may not adhere to ART, one provider stated,* “Some say, ‘I went to a funeral. I was not feeling well or I was just somewhere,' but the side effects of the medicines also contribute. For example, someone may feel dizzy, have bad dreams after taking the medicines, however, when they do not take the medicines, they have good dreams, and so they just decide to quit taking the medicines. They come back to us later when they start feeling sick again.”*

### 3.4. Staff Shortages

All of the healthcare providers cited staff shortages as a factor contributing to nonadherence and provision of ART. As illustrated by the quotes below, some of the providers felt that lack of staff caused them to overwork and turn patients away and ask them to come back another time.* “The main challenges that we usually meet is [sic] workload, as I have already said, we do not only cater to our catchment area, we [sic] also cater to some patients who are coming from other places, so the staffing levels cannot cope with the numbers of patients that are coming in. We have few staff, many patients, and we have few doctors especially the consultants. We need more space and staff.”* Every interviewee gave similar responses about lack of staff and many patients, which increased the wait time to be seen.* “So the problem that we face is that sometimes when patients come they find a lot of patients before them and they think that they will not be helped….”* Another stated,* “There are some problems that we face, these are because we do not have enough staff. We have few staff, while people who need to be helped are many.”*

### 3.5. Drug Shortages

Drug shortages were another issue cited by four healthcare providers as affecting adherence. For example, one provider stated,* “The second issue is the erratic supply of drugs, sometimes you find that some drugs are not available. We are forced to go and borrow from somewhere else or we send patients back telling them to come on another day, that's another challenge.”* Another stated,* “The problem that we are facing is mainly lack of medicine because these ARTs are different types so it might happen that we do not have other regimen available.”* This has forced healthcare providers to have to go to other ART clinics to borrow drugs for their patients.* “And there are times when we run short of drugs. So we have to chase for drugs to other centers that have the particular drugs we do not have here.”*

### 3.6. Belief and Knowledge of ART Outcomes

Belief and knowledge of ART outcomes were identified as both positive and negative factors impacting women's adherence behaviors. Allinterviewees cited patients' belief and knowledge of ART outcomes as one factor negatively and positively influencing adherence to ART. According to participants, some of the patients' believe that ART could cure them of HIV/AIDS, which has increased adherence to ART. Below are quotes in which providers explained patients' perceptions of ART outcomes.

*“When they take the medicines for a long time and see that their body is healthy and they are recovering, compared with the first time when they were skinny or weight less than normal. When they come back to the clinic and find out that they weight more than before and are looking good as if they are not HIV positive, they have faith that they have been cured.”* Another provider stated,* “They think ART are for cure, they think the medicine could cure them of HIV.”* One of the providers stated,* “A lot of patients believe they are good medicines compare [sic] to the time when they started taking the medicines. Now they are differentiating, some say they are able to work now.”* He further expounded on what some of the patients said to him when they come back for refills.* “While at the beginning, we could not do anything to help ourselves, ART is helping us in different ways. We could now go farming, we are now doing any job at the house, and we are not experiencing any problem. While at the beginning, before we started taking the medicines, we had a lot of problems, we could not help ourselves; we were only depending on other people to help us.”*

Some beliefs and perceptions can negatively affect adherence to ART. Below are quotes illustrating how beliefs could affect patients' adherence to ART.* “Because some have been taking the medicine for a long time, they have gained weight compare [sic] with before they started taking the medicine. When they see that they are healthy, they just stop taking the medicine. They think that they are completely cured.”* Another provider stated,* “Some patients do have the perception that after taking the medications for some time they are supposed to stop, because we do routine viral load monitoring and when we tell them the results of their blood sample, they are very happy that the viral load was lower than detectable level. The patients feel that implies the medications have cured them of the disease. They have the mentality that if they are taking their medications as directed, a time would come when they could completely stop taking medications. That's the perception that some patients have.”*

### 3.7. Religion and Adherence

Majority of providers stated that religion contributed to lower or nonadherence among patients because patients were either encouraged to stop taking their ART after being prayed for at their churches or stopped their ART completely during fasting (Muslims). The following quotes from providers explain the impact of religion on adherence: “…some* patients can just choose to miss medications for no apparent reasons at all or sometime after going for prayers. Majority of women nowadays like going for prayers and after being prayed for, they are encouraged that maybe they are delivered from the illness that they had and can stop taking the drugs for a while*.” Another provider stated, “*the Muslims, they have adherence problems, let us say during their fasting month they skipped medications…we try to tell them, please despite the fact that you are fasting, you should still be taking your medications.*” Another provider at the QECH ART clinic stated,* “Some will say if they just pray harder they could eventually be healed and not keep taking the medications, because taking the medications is a problem for them so they think with prayer they could get healed and stop taking their ART medications.”*

### 3.8. Patient Education

Five providers reported that education is a useful approach to increasing patients' adherence. Participants cited that they teach all patients who come to the clinics for medication refills about proper ART guidelines before they give them the medication. This is illustrated by the following quotes:* “Every teaching happens before they get the medicines, they follow the ways that we taught them. What helps sometimes is…we sit and discuss with them the benefits of coming here at the right time, the benefits of taking their medicines at the right time.”* Another provider stated,* “When they start their medicines, we put them in groups where we teach them the importance of taking their medicine according to the prescription.” *Providing patients with education on ART outcome and proper usage seems to increase adherence.* “That's why I am explaining this, 95% is coming because we taught them what they need to know and we gave them the right advice. We teach them the importance of taking their ARTs at the right time and also the dangers of not coming to the hospital to get the medicines.”*

### 3.9. Supportive Network

According to all of the interviewees, more adherent patients have some form of support in the home, hospital, or among their friends, compared with nonadherent patients. Patients' support network includes a formal ART support group who is trained to assist current ART patients on adherence and family and friends. The influence of both types of support on patients' adherence behavior is illustrated in the following quotes:* “When they start their medicines, we put them in groups where we teach them the importance of taking their medicine according to the prescription. Therefore, every group is doing well. I think for these patients to take their medicines according to the prescription, it is because of the help they get from the guardians and support groups in different villages. Those support groups are the ones that are really helping. In support groups, they make sure that they spread the importance of coming to the hospital.”* Another provider stated,* “Since in the group counseling session each client comes with a guardian, the guardian is also influential in terms of patients ART intake.” *One provider stated,* “before the client is started on ARTs, they undergo a group counseling session where many more messages are relayed to them, where they can ask questions. Also in the course of seeing them, we have an individual counseling session where we strengthen them about adherence and find means and ways on how they can adhere to treatment.” *When asked who they believe influences patients' adherence behavior, one provider stated,* “I believe community leaders, friends, and relatives are major influencers in patients ART adherence behavior.”*

### 3.10. Patients' Counseling

Providers focused on the use of counseling as a key approach to improving adherence to ART medication among patients with HIV. Patient counseling was seen as relevant in aligning patients' adherence behavior with providers' expectations. When asked what measures are in place to help patients adhere, one participant stated,* “The measures that we put in place are HIV counseling sessions, pre-treatment and during treatment. Counseling sessions seem to be successful.”* Another provider stated,* “…we are able to notice that this patient is not adhering to medications. The moment we notice that, we do one-on-one counseling as a way to find out the reasons why the patient is not adhering to medications. Sometimes we do it, if it is not a bad case, a clinician does the counseling in the consultation room, but if it is a worse case, we ask the counselors to have a separate session with that particular patient to work out the modalities on how they can adhere to medications better.”* Providers use individual counseling sessions as a means to strengthen patients and help them adhere to medication, as stated by one participant:* “we do have an individual counseling session where we strengthen them about adherence and find means and ways on how they could adhere to treatment.”*

### 3.11. Nutritional/Food Supplements

Some of the participants described strategies to manage patients' nonadherence to ART. One such approach is providing the women access to food (nutritional supplementation). Some participants perceived that providing patients with food supplements would help improve their adherence to ART. One participant talked about the need for food/nutritional supplementation in order to help patients adhere better.* “The other challenge is that some of our patients are malnourished; they come in and they are supposed to start ARTs but you find that their body mass index (BMI) is very low, but we do not have a provision for nutrition supplements. Previously, we used to have the nutrition supplements but these days the nutritional supplements had ceased coming to the ART clinic, so we just tell them you should be preparing your food like this. Which might not be feasible to the patients, other than maybe if we were providing supplements ourselves to them, it could improve their health status faster than when we want them to use their own food [sic]. We are providing them with drugs but we are not providing them any nutritional supplement.”* When asked what they think they need to provide better services to patients, one provider stated,* “the Ministry of Agriculture can help us provide therapeutic feeding to the women, which will help them adhere more.” *Another participant stated,* “I have already said that some patients do not take the medicines frequently on a daily basis as advised because they do not have enough food…back in the days, we were able to give them therapeutic food, but we do not give them the food anymore. If they could also help us to provide therapeutic food to these women, that would be good. We are able to tell that some of our patients need this therapeutic food to adhere better.”*

## 4. Discussion

Antiretroviral therapy (ART) is necessary for alleviating the global impact of the HIV/AIDS epidemic. This study highlights pertinent insights from different healthcare providers on factors relating to antiretroviral adherence and nonadherence in patients with HIV/AIDS. All participants identified barriers that could limit them from effectively providing ART to patients. Therefore, it is important that the implications of these findings be considered when developing effective multidisciplinary strategies for improving ART adherence.

Our findings indicated that the majority of providers have some beliefs about the barriers and facilitators of adherence. We will discuss providers' beliefs regarding (a) reasons for patient nonadherence, (b) environmental factors supporting patient adherence, (c) structural factors related to the clinics which interfere with adherence, and (d) strategies for improving adherence.

### 4.1. Reasons for Patient Nonadherence

According to the theory of planned behavior [21], patients'* attitude *toward treatment adherence includes evaluative opinions of patients regarding whether outcomes of a behavior (ART adherence) could be positive or negative [22]. In this study, healthcare providers stated that the majority of patients had a positive perception of ART outcomes, which influenced their adherence behavior. The notion that an individual's positive attitude toward a behavior is correlated with its practice is supported by a previous study on medication adherence [23]. Ajzen [21] stated that patient knowledge regarding a health behavior shapes the foundation for patients' outcome expectancies, and the significance patients placed on these outcomes forms their attitudes toward the behavior. In this study, providers stated that most patients had some knowledge of ART outcomes and an understanding of procedures for taking the medication. However, providers also stated that some of the patients did not take their medication if they had not eaten beforehand, which suggests patients believed that lack of food was a barrier to ART adherence and that lack of food will make it more likely for them to experience unwanted side effects and more hunger. Similar beliefs were observed by Weiser et al. [24], who discussed mechanisms by which food insecurity led to ART medication nonadherence. Some of the mechanisms Weiser discussed were that medication side effects were worsened in the absence of food and that the medication increases appetite and causes intolerable hunger in the absence of food. In our study, it appears that healthcare providers stated the women were being given misinformation regarding eating before taking their medication. However, providers did not identify who gave the women the wrong information. On the other hand, healthcare providers said they informed the women about following proper medication guidelines. The misinformation given to those women appears to be affecting their decision-making about eating before taking their medication, even though their providers try to disabuse them of the idea. The idea of not taking medication at all due to lack of access to food is problematic for alleviating HIV/AIDS, since many people in developing countries such as Malawi may struggle to obtain regular access to food. Therefore, this finding suggests that, in addition to nonadherence of patients with food insecurity, healthcare providers should also be the focus of intervention efforts since they are the ones who educate patients about the proper guidelines for taking ART.

Beliefs and knowledge about ART outcomes developed from patient experiences with the medication, as stated by the providers. Reda and Biadgilign [25] found that patient beliefs about the positive impact of ART medications on patients' quality of life were associated with good adherence. However, some of the beliefs and knowledge of ART outcomes negatively affect patient adherence to the medication, since the providers stated that some patients would stop taking their medication after they noticed improvement in their health or thought they were cured of the disease. This problem can be prevented by healthcare providers emphasizing the positive beliefs held by patients while providing them with further knowledge and education on the importance of continuing the medication regardless of one's improved health. Providers need to make clear to patients that simply feeling better or having an undetectable viral load does not mean that they are cured [26].

An important contributor to ART nonadherence which providers identified was fear of disclosing HIV status because of stigma associated with the disease. The perceptions that disclosing one's HIV status to others, especially significant others, will cause the spouse to leave them have led some women to avoid taking their ART in the presence of their spouse and, thus, to end up missing doses. These findings highlight the importance of developing stigma reducing interventions that include educational sessions for spouses of HIV-positive patients. In addition, providers reported medication side effects as factors hindering adherence to ART, which is in line with other findings [27, 28]. Thus, there is also a need for discussions about changing the medication regimen to one with fewer side effects.

### 4.2. Environmental Factors Supporting Patient Adherence

Providers identified themselves, along with peers, family members, and other ART patients, as facilitators in patients taking their medication. Providers saw themselves as assisting patients to remember their refill appointment date, encouraging them when they take their medication as directed, and teaching them about the importance of coming for a refill on the date given. They also stated that peer support from other patients on ART, along with guardian/caregiver support, family support, and social support from friends, is essential and influential in helping patients adhere to their medication. It has been shown that good social support helps HIV-positive women handle stigma and discrimination better [29]. Peers are part of the patient support groups which meet regularly to discuss the medicines, how patients can take care of their bodies, and what they can do to live a healthy life with HIV. Guardians and family are responsible for going to the clinics to pick up a patient's medication when he/she is too sick or unavailable to go for refill appointment and remind patients when to take their medication. This finding highlights the importance of supportive networks for improving the health of HIV-positive individuals by increasing adherence. A previous study shows that patient support systems improve adherence and are factors that influence patients to follow advice and instructions [30]. Being a part of a support group gives the women a sense of belonging and helps them reverse the shame and stigma associated with HIV/AIDS.

### 4.3. Structural Factors Related to the Clinics Which Interfere with Adherence

Staff shortages played a major role in provision of ART to patients, which can indirectly affect adherence. Due to understaffing, many of the providers complained about being overwhelmed by the number of patients they see per day and not being able to take a break or annual leave. Staff shortages affect the amount of time healthcare providers devote to discussing ART treatment with patients. These findings corroborate findings by Malta and colleagues [31] on medication adherence, in which they concluded that, due to staff shortages, physicians devoted limited amounts of time to discussing treatment related issues with HIV-positive patients. Drug shortages also played a major role in patients' adherence to ART, forcing healthcare providers to seek medication from other health centers, which in turn affected the number of pills dispensed to their patients.

### 4.4. Strategies for Improving Adherence

Regarding strategies for improving adherence, participants recommended the use of group and one-on-one counseling sessions to help determine what causes patients not to adhere to medication protocols. Counseling sessions would also provide patients with better understanding of HIV/AIDS, ART medication, and proper medication procedures. McPherson-Baker and colleagues [32] found in their study on adherence that providing medication counseling and behavioral interventions to HIV patients increases adherence. Providers recommended the use of food supplementation/therapeutic feeding to eliminate nonadherence to ART, which is consistent with a previous study [33]. Cantrell and colleagues found in their pilot study of food supplementation to improve adherence to ART that food supplements were associated with better adherence of HIV patients in Zambia [33]. Some patients stop taking their medication because of lack of access to food and their perception of side effects associated with taking medication without food, which is supported by other studies [34–36].

## 5. Limitations and Strengths

There are several limitations to this study. We assessed a sample of providers taken only in ART clinics in southern Malawi, which limits the generalization of the results to other healthcare centers with fewer HIV patients and providers without specialized training in treating HIV patients. In addition, we used only interview data for this study, so future research should include observation of participants, patients, and focus group discussion of both providers and patients to assess differences in factors they perceived influencing adherence.

Although the study has some limitations, we believe its strengths outweigh the limitations. One strength of this study is the variability of providers who were interviewed, which increases the chances of having diverse perspectives on factors influencing patients' adherence. The results that were obtained from this study may further our knowledge about factors that influence HIV medication adherence among HIV-positive women from the providers' perspective. The study also helped us understand other possible factors (drugs and staff shortages) outside of patients' control influencing adherence.

## 6. Implications to Health Education and Research

These findings and providers' suggestions have broader implications for other ART clinics in Malawi and future research. The findings highlighted the relevance of healthcare providers' beliefs about their patients' ART adherence behavior in promoting ART adherence. Based on the findings from this study, interventions focusing on increasing collaborations between providers from different ART clinics and their patients will be useful in increasing information sharing between providers at different clinics, patients, and faith-based entities. In regard to the impact of religion on adherence, health educators could collaborate with imams to develop programs focus on increasing adherence during fasting. Providers should be trained and have access to resources that allow them to refer patients to nearby treatment centers when they run out of drugs. Collaboration between providers, community-based organizations, and the government of Malawi could help with the provision of support for adherence. Such support includes supplying food to undernourished patients or assisting with communal gardens for HIV patients, providing treatment centers with extra medication and larger space for dispersing ART.

Healthcare providers' perception of how drugs and staff shortages affect adherence needs further study. This area needs more clarification since it affects the capacity of patients who could be cared for at each treatment center and the amount of time providers devote to one patient. Another area that needs further exploration is women's perceptions of being cured and having improved health after taking ART for a long period, since this affects patients' willingness to continue taking their medication. Based on these findings, there may be opportunities to propose supporting medication adherence at the patient and provider levels as stated by previous researchers [37]. In order for HIV-positive individuals to benefit from HIV medicationsand subsequently lower their viral load, full adherence to treatment is crucial [38, 39]. Improving HIV patients' adherence requires the involvement of patients, providers, and patients' families and friends. We found that providers need more training and supportive supervision of their work. Providers could help by providing patients with adequate information to keep them knowledgeable about ART adherence.

## Figures and Tables

**Table 1:** Providers characteristics (*N* = 8).

	*N*	%
Gender		
Female	4	50.0%
Male	4	50.0%
Age		
20–30	5	62.5%
31–41	3	37.5%
Years working with HIV/AIDS patients		
1 to 6	2	25.0%
7 to 10	5	62.5%
10+	1	12.5%
Occupation		
Nurse	1	12.5%
HIV counselor	3	37.5%
ART clerk	2	25.0%
Other	2	25.0%

Other: clinician and doctor.
